# Health care professionals’ experiences of how an eHealth application can function as a value-creating resource - a qualitative interview study

**DOI:** 10.1186/s12913-021-07232-3

**Published:** 2021-11-05

**Authors:** Catharina Carlqvist, Heidi Hagerman, Markus Fellesson, Mirjam Ekstedt, Amanda Hellström

**Affiliations:** 1 Karlstad Business School, Karlstad University, Karlstad, Sweden; 2 Faculty of Health and Life Sciences, Linnaeus University, Kalmar, Sweden; 3 CTF Service Research Center, Karlstad University, Karlstad, Sweden

**Keywords:** Chronic conditions, Co-production, eHealth, Health care professionals, Inequality, Telemonitoring, Value, Value destruction, Value recovery, Qualitative study

## Abstract

**Background:**

The number of patients with one or more chronic conditions is increasing globally. One strategy to achieve more sustainable care for these patients is by implementing use of home-based eHealth applications. Such services support patients to take on a more active role as value-creating co-producers of their own care, in collaboration with health care professionals. Health care professionals have a key role in the value creation process, but little is known about value formation within eHealth interactions, especially from their perspective. Therefore, this study aimed to provide a deeper understanding of how an eHealth application can function as a value-creating resource from the perspective of health care professionals.

**Methods:**

Semi-structured interviews were conducted with thirteen health care professionals (nurses, physicians and first-line managers). Qualitative content analysis was used to analyze the interviews.

**Results:**

The findings indicate that value formation processes are strongly influenced by the organizational preconditions and by the usability and functionality of technology. The experiences of the health care professionals indicated that value was conceptualized in dimensions of meaningfulness, building of relationships, building safety and feelings of trust. Although these dimensions were mainly expressed in a positive way, such as perceived improvement of medical care, accessibility and continuity, they also had a negative side that caused value destruction. This was primarily due to patient difficulties in using the application or making measurements. Subsequent efforts at value recovery resulted in value creation, but were often time-consuming for the professionals.

**Conclusions:**

This study contributes by extending conceptualizations of value to the role of health care professionals and by highlighting technology as sometimes facilitating and sometimes hampering value formation processes. The findings indicate that the eHealth application was a value-creating resource, facilitating proactive communication and supporting patients’ engagement and control over their self-care. However, for the application to become a more valuable resource in practice and counteract inequity in care, it needs to be further developed to be adapted to the needs and preconditions of patients.

## Background

The number of patients with one or more chronic conditions is increasing globally, which creates a major challenge for already financially strained health care systems [1]. Chronic conditions require continuous management [2] and multimorbidity coordination between different care providers is often needed [3]. One cause of major expenses for society and great suffering for individuals is insufficient observations of vital signs, which can lead to failure to notice deteriorations that could have been prevented [4]. One strategy to achieve more sustainable care for patients with chronic conditions is through the implementation of homebased eHealth services. eHealth is a broad term and is defined by the World Health Organization as *“the use of information and communication technologies (ICT) for health”* [5]. eHealth services in the form of self-monitoring using telemonitoring devices have been shown to decrease delivery costs [6] and improve medical quality [7, 8]. Studies have proved that eHealth has the potential to stimulate interactive communication and enhance involvement of patients in their care [9, 10].

The use of eHealth has increased considerably during the COVID-19 pandemic and digital tools have been shown to be invaluable for changing ways of working [11]. eHealth also offers support to stimulate self-care processes as health care professionals (HCPs) can provide patients with a knowledge-building tool that has the potential to help them incorporate positive health behavior changes in daily life at home [12]. For HCPs, enhanced access to patient data through remote information sharing enables early detection and timely response to deterioration events. This was significantly associated with reduced hospitalization [8, 13] and hospital re-admission rates compared with conventional care [13]. It also enabled early detection of deterioration in patient health that signaled they were in need of hospital care [8].

eHealth solutions support the current transformation of health care delivery from a view of patients as mere passive consumers of care to seeing them as capable of undertaking a more active, responsible role as co-producers in their care management [14–16]. Co-production in health care is a reciprocal collaborative process in which HCPs and patients work together to produce the care service, as well as sharing information and setting goals together to achieve beneficial outcomes, i.e. creation of value [16–19]. Value is conceptualized as individually determined, based on the perceived benefits realized by the parties involved rather than embedded in the features that the eHealth service offers. Instead, value emerges and is formed during interactions and activities in which resources, such as knowledge and skills, are integrated and used by all the actors involved in care [20, 21]. The experience of value is influenced by the unique context in which health care takes place (cf. [22–24]). Therefore, each individual involved may assign a different meaning to the same eHealth service, depending on the situational factors at hand. It is presumed that even if individuals are provided with identical services, they may choose to use them differently to gain value [15]. Although insight within health care regarding the importance of the patient’s role as an active and informed co-producer has increased in recent years [16], patients often lack the means and ability to apply self-care knowledge. As pointed out by Spaling et al. [25], self-care activities have to be adapted to each patient’s unique situation and should provide the patient with a sense of control. Importantly, technology offers flexibility in the HCP-patient relationship, facilitating adoption of person-centered care [26, 27], which is essential for the management of chronic conditions [28].

Value is created iteratively, as resources are continuously created and integrated, throughout the service process [29]. Accordingly, co-production through home-based self-management could result in synergistic benefits for all actors in health care [30]. Further, technology that supports interactions and communication increases opportunities for service providers to create value together with their customers (i.e., patients) [31], as long as they share overall goals [20] and beliefs [29]. The parties’ expectations on the eHealth service and on each other are also of significance for how they will act and how value will be perceived [32]. As highlighted by Batalden et al. [16], co-production also assumes that patients are willing and capable to participate and take on more responsibility, which cannot be taken for granted. Another barrier to successful co-production is the existence of a professional-centric health care culture that is resistant to change, leading to care being provided as a commodity rather than as a service.

However, there is also a potential dark side to co-production of health care services. If resources are not integrated and used successfully, the outcome may be negative. As a result, value can be destroyed or diminished, leading to decreased well-being for at least one of the parties involved [20, 32]. Value destruction can occur unintentionally if parties attempt to co-produce without having sufficient ability, for instance when technology is difficult to use [33, 34] or does not function properly [35, 36]. Further, value destruction may arise from intentional misuse of resources such as underuse or overuse of the services provided [37]. Fortunately, these undesirable consequences may be reversed by value-recovering interactions initiated by the parties involved (i.e., HCPs or patients) [38]. This implies that inherent to the overarching value formation process, there is a dynamic interplay between potentially co-occurring value creation and destruction. By applying a service-oriented perspective of value, where the patients’ experiences are in focus, their position in care can be strengthened. However, while previous research within the service field has addressed this interplay from a patient’s perspective [14, 15], neither health care nor clinical work can be reduced to solely a matter of patient experiences and perceptions [16]. A comprehensive understanding of health care value formation dynamics requires that, e.g., medical, technical, ethical and organizational, work-related aspects of the care situation and the health care service at hand are also captured. The professional perspective of HCPs constitutes a natural starting point for this.

Earlier research has described HCPs’ experiences of value destruction when using eHealth services. For example, in a recent interview study [39], nurses described a perceived lack of time and that they did not have the digital skills necessary. They also expressed concern about adjusting to new roles and the risk of losing their role as experts in advising patients. Lie et al. [26] found that asynchronous communication caused the nurse-patient relationship to become more fragile, and nurses raised concerns about the risk of misunderstandings. On a positive note, openness was facilitated and nurses also experienced a better understanding as a result of reading messages sent by patients [26]. According to the Swedish eHealth strategy [40], eHealth is expected to reduce inequities and inequalities in health by making care accessible to the entire population, regardless of geographic location and time. However, problematization on how to close care gaps in vulnerable patient groups is scarce [41]. In fact, as revealed by HCPs, insufficient ability to use the technology, due to either patients’ impaired health or low technical competence may create new health inequities and inequalities [42], and result in value destruction.

In summary, the implementation of eHealth has resulted in both positive and negative outcomes for HCPs and patients. However, little is known about how value is formed within eHealth interactions and the HCP perspective has been neglected, when it comes to how HCPs view both the patients’ value-creating role and their own contributions. Therefore, it is of interest to study the value formation process in eHealth from a HCP perspective, as successful implementation of digital services requires that everyone involved is motivated in their work and supported by an engaged leadership [43]. In order to add knowledge on these issues and to address the role of technology in the value formation process, this study aims to provide a deeper understanding of how an eHealth application can function as a value-creating resource from the perspective of HCPs.

## Methods

### Design

We conducted a descriptive study with a qualitative approach. This study was a part of a telemonitoring project that focused on increasing safety, security, participation and continuity in care, as well as to reduce the number of emergency visits in patients with chronic conditions. In this pilot study, we focused on co-production of care between chronically ill patients and out-patient care facilities through the use of telemonitoring.

### Setting and sample

The study was carried out in a region with approximately 250,000 inhabitants, located in the south of Sweden. It has one county hospital, two district hospitals, and 39 health care centers (HCC) providing health care service at the primary and secondary level to inhabitants. Four public primary HCCs and one medical department at one district hospital were selected by the region to pilot the eHealth service. The primary HCCs were located in urban and rural areas. All HCPs (seven nurses, two physicians and four first-line managers (FLMs) who worked directly or indirectly with the telemonitoring at the selected units were asked to participate in the study and all agreed to participate. The participants received written and verbal information about the study aim and procedures. All participants gave their written informed consent to participate in the study. See Table 1 for participant characteristics.

**Table 1:** Characteristics of participants (*n *= 13)

**Age in years**, md (range)	49 (28–59)
**Gender**:	
- Female, n	12
- Male, n	1
**Profession**	
- Nurse, n	7
- Physician, n	2
- First-line manager, n	4
**Years of experience**	
- Nurse, md (range)	13 (6–39)
- Physician, md (range)	26 (19–33)
- First-line manager md, (range)	8.5 (8–11)
**Years at the workplace**, md (range)	4 (1–30)
**Employment status**	
- Full-time, n (%)	10 (77 %)
- Part-time, n (%)	3 (23 %)

*md *median, *n *number

### The telemonitoring intervention

The region was interested in testing two different eHealth services for remote health control (telemonitoring) of chronic conditions in primary care (for further details, see Table 2). The eHealth services could be used by patients on a tablet or smartphone. The patients needed to have sufficient cognitive and functional ability to use medical devices such as blood glucose or blood pressure monitors, and to understand the implications of the monitored health data (physiological measurements). Patients with heart failure, hypertension or diabetes had access to the telemonitoring applications and medical devices for six months. Patients who did not have the abilities needed to use the technology were dependent on support from relatives. The patients reported health data from their private homes in accordance with their individual plans set up with the physicians. Data transmission was performed manually and/or by automated technical means. The patients’ health data were transferred to the care departments, over a secure internet connection. Nurses at the care departments monitored and registered the data in the respective patient’s electronic health record. The nurses also kept in contact with the patients via chat or video meetings through the telemonitoring applications.

**Table 2:** The telemonitoring applications^a^

	Telemonitoring application 1	Telemonitoring application 2
**Number of departments using the application**	2	3
**Patient diagnosis**	Heart failure	Heart failure, diabetes, hypertension
**Patient generated health data**	Blood pressure, heart rate, weight, oxygen saturation, temperature	Blood pressure, heart rate, weight, oxygen saturation, temperature, physical activity, blood glucose level
**Functionalities**		
- Chat	Yes	Yes
- Video meetings	Yes	No
- Alerts	No	Yes
- Support from distributor	Support for HCPs, not for patients	Support for HCPs and patients
- Medical devices	Stationary in patient homes	Mobile
- Ability to send images and links	No	Yes, for HCPs
- Integrated with the medical record system	No	No

^a^Web portal application for HCPs and mobile application for patients

### Data collection

Semi-structured interviews with the nurses and physicians were performed between March and May 2020 and with the FLMs between October and November 2020. Due to the COVID-19 pandemic, all interviews were performed via Skype, Zoom or telephone. All interviews were recorded and lasted between 27 and 86 min. The interview guides consisted of open-ended questions focused on describing experiences of the use of a telemonitoring application at work, organisational preconditions, work satisfaction, partnership and cooperation with the patients as well as safety and security aspects. Information probes were used to clarify participants’ experiences, examples include “please tell me more” and “can you give an example”.

### Data analysis

The interview data were analyzed with qualitative content analysis guided by Graneheim and Lundman [44]. According to Krippendorff [45], qualitative content analysis is a suitable method to use when manifest and latent contents in an interview are to be analyzed systematically. The analysis was performed using NVivo version 12 (QSR International Pty Ltd). First, the interviews, transcribed verbatim, were read several times, to get familiar with the text. Then, meaning units that contained sentences with related content based on the study aim were extracted and labelled with codes. The codes were sorted into five subthemes based on differences and similarities. These subthemes were: *“The importance of having sufficient preconditions,” “The telemonitoring process,” “Perceived value outcomes,” “Value recovery,”* and *“Continued development of telemonitoring for eHealth.”* The subthemes were abstracted into one theme, *“The struggle in the value formation process,”* which describes the thread of meaning that runs through the codes and provides a description of the value formation process of telemonitoring (Table 3). The analysis was a dynamic process, where the entire research team was engaged in discussions to ensure that trustworthiness was achieved [44].

**Table 3:** Examples of the analysis process

Meaning units	Codes	Subthemes	Theme
“It has given me more work, I feel, because I have to check every day. And before I didn’t have this daily contact or check-ups of these patients, not at all.”	Telemonitoring creates extra work	The importance of having sufficient preconditions	The struggle in the value formation process
“So I have been monitoring these measurements and that’s been basically every day and that together … that the patient provides blood pressure and that you can easily communicate with the patient through the chat, has been very good in … And they have been able to contact me at any time and I’ve been able to respond and that kind of thing. That’s … I think it’s been great.”	Digital communication with patients	The telemonitoring process	
“It’s nice when you feel that you can help and that you can help in time and that you prevent a potential admission and that they can stay at home.”	Meaningful work task	Perceived value outcomes	
“Yeah, but sometimes you get the ones that are … totally wrong, like a weight of 26 kilos, maybe, or something, and then you have to interpret that as a … like a completely erroneous value and ignore it. And then I can write a message to the patient, that I see that your weight is very different today, could you measure that again, and so you solve it that way. Or it was the grandkid who tried the scales or whatever it might be. So … but otherwise I just contact the patient and ask them to make a new measurement.”	Incorrect data requires me to act	Value recovery	
“And then maybe you need to have it during 3–4 months or half a year or something, that you have it more for training purposes or examination purposes or … yeah, I think that you need to, like, maybe personalize it, like who is supposed to, in a different way.” [Hypertension]	New areas of usage	Continued development of telemonitoring for eHealth	

## Results

### The struggle in the value formation process

**Fig. 1:**
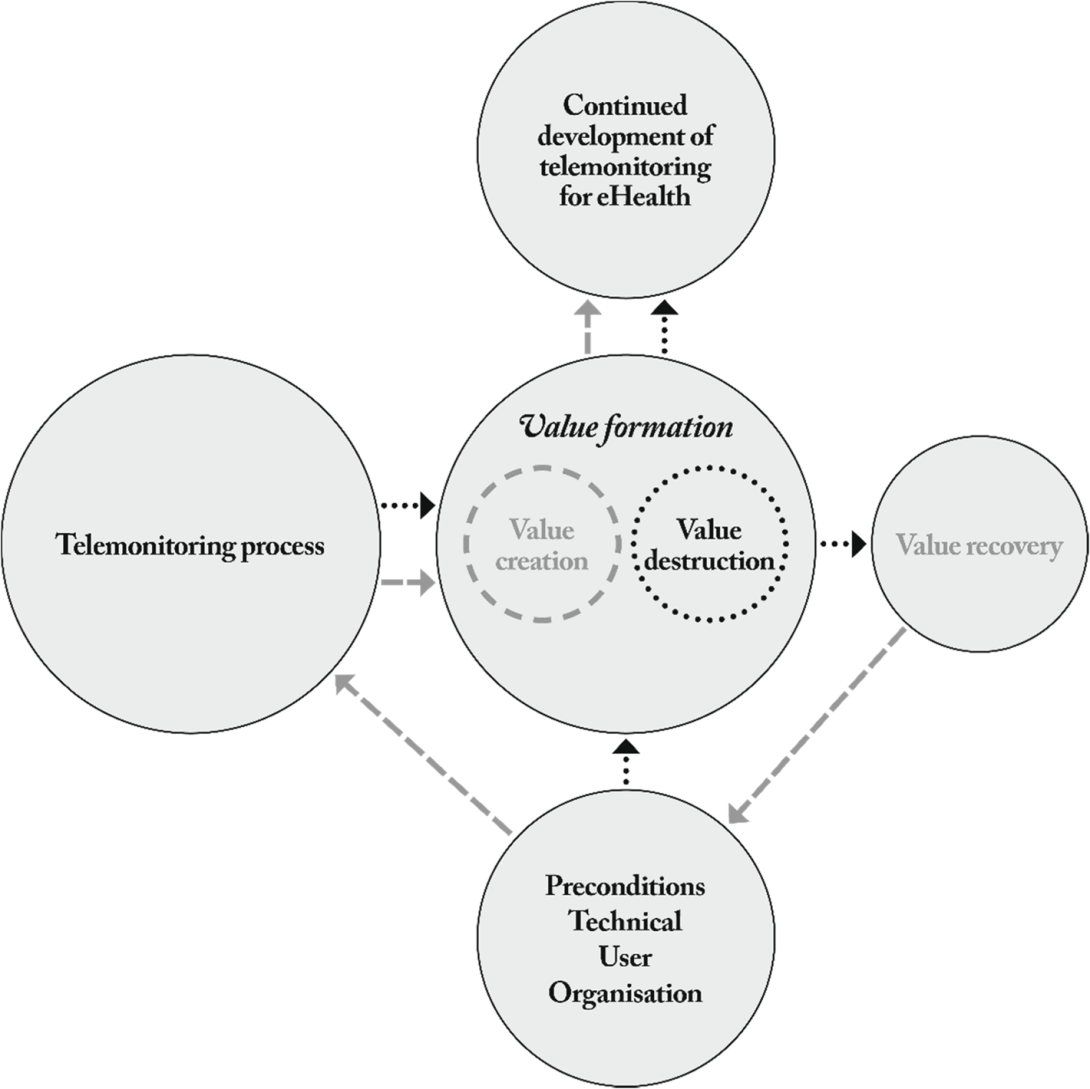
The value formation process. Dotted lines and arrows = value destruction. Dashed lines and arrows = value creation

The results from the study are here presented and discussed thematically, with reference to Fig. 1, which relates the different subthemes to each other and addresses telemonitoring value creation at an overall level within the theme: the struggle in the value formation process. This theme describes the HCPs’ experiences of working with a telemonitoring application to create value in care with patients as active participants. The value formation process can be illustrated as a circular process encompassing the preconditions, the monitoring process and the creation or destruction of value. To achieve success, sufficient preconditions in the organisation, for the users and the technical application (bottom circle in Fig. 1), were of great importance – as was interactive communication between the HCPs and the patients in the monitoring process (left-hand circle in Fig. 1). When the HCPs described how they experienced their interactions with patients and technology, four value dimensions that could have both positive (value creation) and negative aspects (value destruction) were identified (central circles in Fig. 1). These dimensions were: meaningfulness, building of relationships, building safety, and feelings of trust. However, recovery efforts (right-hand circle in Fig. 1) by the nurses and patients through interactive communication could counteract value destruction, resulting in creation of value. Each value outcome then becomes part of the preconditions for future value formation processes. Knowledge gained by the HCPs during the value formation process also informs continued development of telemonitoring for eHealth (top circle in Fig. 1).

### The importance of having sufficient preconditions

#### Organisational preconditions

The organisational preconditions that were of importance were described in terms of time, support, technology, and usability. Having sufficient time for work was emphasized. Some nurses experienced that the daily readings of health data increased their workload. In contrast, a few nurses said that the time allocated was adequate and that monitoring had not taken much time. This may depend on whether or not their FLMs had scheduled enough time to work with telemonitoring. However, the FLMs did not get any extra time to work with the project. The perceived lack of time was mainly related to solving problems with technology, either in the patients’ home or at work. The HCPs described support from colleagues, management and the technology company as essential.

“*Make sure that you have good help from the support and that the entire group are aware that this will take time and that you have an open dialogue with the entire care center about what they are doing, so that there isn’t some … Oh, now they’re at it again with that project with those two. You have to be open about taking time and that it takes resources.*”

However, support was sometimes lacking. For example, nurses described a lack of individual care plans for the patients, which affected their work negatively. This was of particular significance in workplaces with temporary physicians hired. The system was described a vulnerable, because only a few people were involved. As a solution, a central unit was suggested as backup. The HCPs called for the technology company to provide patients with support, instead of having the nurses make home visits. Working with telemonitoring was described as time-consuming due to its shortcomings. The telemonitoring applications were not integrated with the digital medical record system, which was a major obstacle to an efficient way of working. Another major obstacle in application no 1 was the lack of alerts signaling deviating measurements. In contrast, nurses working with application no 2 got a lot of alert notifications when they started using the system, which was difficult to handle.

#### User preconditions

The users, both HCPs and patients, had to have some technical skills to be able to work with the application. In the pilot project, the nurses were chosen based on familiarity with technology, in order for them to feel comfortable. Some nurses chose not to participate due to low digital literacy. One weakness described was that nurses were asked to help patients replace batteries or start devices that had been turned off unintentionally, which caused negative disruption at work. Another weakness that affected the nurses’ work negatively was that not all patients had been able to use the chat. Therefore, HCPs highlighted the need for simplified applications with minimal choices for these patients. Overall, the HCPs described the use of the new technology as fun, exciting and motivating.

“*It felt almost like Christmas, that we got to sit there and pick up and, yeah, now we get to do something fun at our unit.*”

#### Technical preconditions

Technical preconditions refer to the eHealth applications and connected medical devices and the extent to which the service could fulfill the needs of the patients. Overall, the telemonitoring applications and medical devices were found to be user-friendly and to work well. However, the nurses described having to support the patients when the applications were difficult to use or when technology was not functioning properly.

“*…then there’s frustration among the patients and they don’t get their values [health data] sent in and then we’ll say something like: never mind, in that case // it’s not really your fault, because there’s something wrong with the machine. Because there’s a lot of frustration over these measurements that are wrong and went all weird.*”

The nurses also described that the telemonitoring needed to be portable for occasions when patients were away from home, otherwise some patients could not benefit from the technology.

### The telemonitoring process

The monitoring process that took place through the application consisted of assessing patient-generated health data, and communication through either direct interactions in video meetings or indirect interactions in the chat. The telemonitoring enabled the patients to monitor and check their own health data and to interact with the nurses. Thus, the nurses became contact persons for their patients, which enabled them to help the patients based on each patient’s individual needs. The video meetings and chat served different functions. The nurses experienced that video meetings created a more personal environment that opened for conversations on all kinds of topics (which were sometimes time-consuming). Video meetings were favored over the chat as they gave the nurses possibility to be proactive and detect visual symptoms and health deterioration in their patients. This was especially important in the case of heart failure patients because of their severe condition. The chat was more focused on the patients’ medical needs, not on the relation between nurse and patient.

“*We had questions at the start, that our patients would use … overuse the chat function and sneak in questions about this and that and … yeah, in a more boundless way, and we haven’t seen that … Instead, it has really been … they’ve sent in the values [health data] that we’ve asked for and not a bunch of other information. And they haven’t overused them to get access to other functions on our side, no.*”

However, analogue communication was sometimes needed. For example, as the nurses only checked on the patients’ health data during office hours, patients had to use the phone to contact their care provider after hours. The nurses also had to call patients when the application was not working or when the patients did not know how to use it. In order to avoid misunderstandings, medication lists were sent by post, instead of being sent through the application for telemonitoring.

### Perceived value outcomes

#### Meaningfulness

Most HCPs experienced that telemonitoring added meaning to work and it was described as a fun, satisfactory and rewarding way of working, without resulting in perceived depersonalization of care. They also found it stimulating that the patients were actively engaged in the care process.

“*Yeah, I think it has been very valuable. I have, in the past, seen eHealth as something kind of negative and thought a lot about that the personal meeting will disappear and you don’t meet face to face and all of that, but I’ve had to change my mind. I think this works really well.*”

The HCPs – nurses, physicians and FLMs – emphasized how health data monitoring had increased their insight into patient health, leading to more proactive and elective care. The physicians described that the patients’ reports of health data made it possible for them to make appropriate drug adjustments without in-person visits. Other reported strengths included reduced needs for regular check-ups and planned care visits to evaluate the effects of drug therapy. However, it was expressed by HCPs that not all patients were in need of telemonitoring, and that some patients only needed the monitoring for a short while, usually when newly diagnosed. It was also recognized that telemonitoring had no value in itself and should not leave the patients feeling sicker than they really are. Creating unnecessary care dependency by tying patients to health care longer than needed was considered undesirable and not meaningful by some HCPs. Another aspect of meaningfulness was that telemonitoring made it possible to observe and check up on the patients’ health measured data. This enabled for HCPs to provide first level of care and empower patients to avoid being transferred to a higher care level. Some nurses stated that the applications enabled them to set goals together with the patients. Both nurses and physicians underlined that the patients gained knowledge of and control over their health, which made it easier for them to understand and practice self-care as well as to be adherent to medication. However, not all nurses had been engaged in setting goals with their patients and it was said that the patients had been given too little information on self-care.

When meaning was described in a larger context, telemonitoring was considered meaningful to society as a strategy to meet future demographic challenges and prevent hospital admissions. When patient knowledge increased and patients took greater responsibility of their health, society could benefit from a more proactive approach to health. Furthermore, it was pointed out that telemonitoring was an effective way of performing regular follow-up of patients with chronic conditions. However, concerns were raised that some patients were given a fast track into health care, which could create inequities and inequalities that could be negative to society.

#### Building of relationships

Another value dimension expressed was the fostering of positive relationships with the patients. The nurses emphasized the importance of continuity and for patients to have direct access to the nurses through the application as this enabled the nurses to collaborate and discuss with patients in a completely new manner. The care relationship was also experienced as becoming more equal. Some nurses stated that they had gotten to know the patients in a completely different and deeper way. This was particularly evident among the nurses who had used video meetings.

“*And we have gotten … well, a lot of existential questions, a lot about death and if you are ready or not and … Relationships, like, in general, between spouses. And you maybe wouldn’t bring that up if you went to see the district nurse at the local care center to get new compression wraps on your legs, then maybe you wouldn’t ask those questions.*”

However, several HCPs emphasized that although digital communication was important, it could not replace all kinds of physical care encounters.

#### Building safety

Due to the perceived reliability of the system, the nurses and physicians felt confident in working with the telemonitoring application and experienced it as contributing to a process of safety building. This was mainly accomplished through the readings of health data, which enabled them to follow the patients’ health data over a longer period of time. Another benefit was mentioned in relation to patients with hypertension: blood pressure values were considered more reliable when taken daily at home, compared with when measured more seldom at the health care center, which could be perceived as a more stressful environment. However, although there were positive effects of having patients making measurements at home, concerns were expressed regarding if the measured data were reliable due to some patients having difficulties using the technology. Still, most data inaccuracies caused only minor concern, as they were obvious. Instead, concern primarily was related to deficiencies in the system that could cause loss of control. This was particularly the case when no readings were made during weekends, holidays, and during on-call hours.

“*Yeah, even during on-call hours, you can get a weight increase or something, and who is keeping track then? So the patient has to keep track of that themself and raise the alarm or, like, know what to do.*”

Furthermore, when the applications failed to transmit health data due to technical problems or when a patient did not understand how to use the telemonitoring applications, this affected safety negatively. However, a telemonitoring system might never cover all eventualities and sometimes admissions are necessary. In two cases, patients were admitted to hospital due to rapid progression of their conditions that could not have been prevented. Another potential risk described by one FLM was related to privacy protection in telemonitoring: where are health data stored?

#### Feelings of trust

Trust is different from safety – as a value it refers more to individual experiences than to the actual reliability of the system. HCPs experienced this value when patients expressed to them that they felt secure and had trust in the applications, knowing that the nurses had responsibility for and kept track of the patients’ health data. On the other hand, this raised concerns about how accessible the nurses should be to the patients. The nurses experienced feelings of trust when the patients became independent and when they could support their patients to become competent and empowered and to contact care providers on their own, when needed.

“*But he felt that it was really nice to have these things at home and I knew that I could check on him as much as I wanted and, yeah, if there was an emergency, well, then he should get emergency care, of course, but he still felt safe in that, that he had the possibility to monitor it at home and he dared to wait and that it passed later … plus that he also knew that there was someone on the other side checking his values [health data]. Not like urgently in the moment, maybe, but the next day at least.*”

### Value recovery

When resource integration failed (i.e. value was destroyed), attempts at recovery were often initiated by nurses or patients. Most episodes that the HCPs reported were related to patient problems in working with the applications or devices. For instance, in case of inaccurately measured data, the nurses sometimes had to send chat messages to the patients.

“*… sometimes you get a weight of four kilos and then you know that either they’ve placed a grocery bag on it or it’s the dog or something. Or a temperature of 26 degrees, so sometimes I’ll write that you’re getting colder, could you check your temperature again? Because then we know that this is so … this isn’t right.*”

Another attempt at recovery occurred when nurses made home visits to help patients with technology-related problems or gave instructions over the phone. Sometimes, the nurse and patient had to interact with the support company to solve problems. It was also described that patients sometimes came by the care center and asked the nurses for help when they had difficulties in using the application. In order to recover value, the patients sometimes send their measured health data by post to the care unit when the technology was not working. One nurse described feelings of helplessness during the recovery process, when unable to solve technology-related problems and that it took time before patients could enter their measured data. In one case, recovery efforts were related to social aspects. In order to compensate for isolation caused by the loss of home health care when telemonitoring was introduced, one nurse provided a patient with social support in weekly video meetings and worked hard to arrange support from the municipality – without success.

### Continued development of telemonitoring for eHealth

On the basis of the HCPs’ experiences of working with the service, several limitations in the application could be highlighted as areas for improvement and future development. Chat messages could not be translated, which excluded non-Swedish speaking patients. Further, the nursing part of the application had not achieved its full potential and some nurses wanted it to be developed further. When the HCPs described potential new areas of use, they mentioned that telemonitoring could be valuable in the management of other conditions that require frequent care contacts and that the application should be developed to support patients with multimorbidity.

“*And I also think that you could use the tablet for other diagnoses as well // And have an easier pathway into care somehow.*”

For telemonitoring to become even more efficient, HCPs suggested providing education to newly diagnosed patients and placing greater emphasis on health behaviors such as diet and exercise.

## Discussion

The aim of this study was to provide a deeper understanding of how an eHealth application can function as a value-creating resource from the perspective of HCPs. This study has contributed with a conceptual model for thinking about how health care value can be both created and destroyed depending on the prevailing preconditions and how value is perceived by HCPs in the dimensions of meaningfulness, building of relationships, building safety and feelings of trust. Unlike traditional service research, our perspective was broadened to explicitly take into account that health is more than just patients’ experiences and that clinical care work consists of an interplay between HCPs, patients and technology [16]. The telemonitoring application provided the HCPs with new digital opportunities to interact with their patients at distance, which has been emphasized as a key aspect in order to enhance the possibilities for value to be created [31]. The use of an application made it possible for HCPs to involve patients in co-production processes, which might contribute to the building of stronger relationships [18] – something that was described as adding meaning in the daily work of the HCPs. This indicated common goals and harmonizing values, which are considered to be necessary preconditions for successful value outcomes [20, 29].

The telemonitoring application functioned as a facilitator of resource integration that also created presence and continuity in the relationship between HCPs and patients. Thus, it contributed to values of safety and trust. This could be particularly valuable for patients living in rural areas as the use of eHealth could enable more regular care contacts. Involving patients as partners contributes to the transformation of care from paternalistic to collaborative and the changed roles between patients and caregivers that follow from that [14–16]. This could be perceived as a threat to the traditional role of HCPs as the sole experts [39]. However, the HCPs in this study felt confident in their counselling role and encouraged patients to develop their ability to take an active and responsible role in reaching their own health goals. Importantly, responsibility was shared and not handed over [46]. It has been seen that use of eHealth has been accompanied by risks of making the nurse-patient relationship more impersonal [26, 39]. In this study, most HCPs experienced trust in this way of communicating and felt that technology did not lessen the human aspects of care. This is contrary to the results of Lie et al. [26], who stated that nurses felt insecure about communicating with patients through written messages. However, it should be noted that our results might have been influenced by the fact that the nurses participating in this study were already accustomed to communicating with patients through technology. The type of care involved (e.g. as regards medical and/or emotional complexity) might also impact the perceived suitability of digital communication.

While value creation is the goal, collaborative value formation processes may be negative and lead to destruction of value for the parties involved [20]. Our findings indicated that eHealth technology played a dual role by acting as both a facilitator and an obstacle to the creation of value, depending on the dynamics of the unfolding value formation process [20]. The studied service also improved accessibility between the parties, which created feelings of trust. However, feelings of trust do not necessarily mean that the care provided is safe. Although the patients were often proactive in their own care, nurses said that they sometimes had to be proactive when the measured data deviated. This underscores the necessity of HCPs taking an active role in the telemonitoring process, as not all patients can manage this on their own [36]. Through the interactive communication, the nurses were also provided with important insights into the patients’ value creation processes in their everyday lives, which are usually separate from the care provider [23]. Though interactions in the chat and video meetings both created value, the nurses stated that video meetings, in particular with heart failure patients, enhanced value creation as the nurses could see their patients and visually detect health deterioration as well as provide these severely ill patients with support. Some nurses said that the use of the application made it possible to react in a timely fashion and prevent patients with unstable heart failure to be admitted to hospital. This was described as one of the most valuable aspects of using the application.

The HCPs said that working with the applications was stimulating and fun, but also resource-demanding and sometimes suboptimal for successful co-production. The FLMs expressed an intention to create good working conditions for the nurses and physicians, for example by having an ongoing, continuous dialogue and enabling flexibility in their work. However, some HCPs stated that they lacked resources in the form of time and support, which negatively affected their working life and the value creation process. Furthermore, lack of integration between systems and poor usability of the technology impaired the HCPs’ experiences, which is consistent with the findings of Öberg et al. [39]. This is a serious problem, as nurses already experience high workloads and suffer dissatisfaction at work [47]. This points to the importance of having sufficient organisational preconditions [48] and of technology being easy to use, so that it contributes to a more efficient and enjoyable way of working.

Other important preconditions to consider are that the technology must be functioning, and that the patients must be able to use the telemonitoring application. If these conditions are not at hand, this can result in destruction of value [34, 35] as well as decreased patient engagement [33], constituting a critical threat to patient safety, which could impede future co-production. With eHealth comes concerns about how to store data safely and securely, to ensure trust in the service. This aspect needs to be further addressed to avoid destructive events. In this study, most reported episodes of value destruction occurred when patients could not use the application or devices. Interestingly, in attempts to recover value, both patients and nurses participated and performed activities to solve and mitigate the negative consequences of such events. These findings are positive, as the results from a study by Dong et al. [49] suggest that joint recovery of value can create higher perceived value for patients and increase their motivation for further engagement.

Turning to a medical perspective, the HCPs suggested that telemonitoring should only be provided to patients who are truly in need of this service. Patients with severe conditions with an inherent risk of hospitalization, such as heart failure, should be offered the service on a continuous basis. For patients with high blood pressure and diabetes, telemonitoring could be more suitable to use for a shorter period of time, for example to support newly-diagnosed patients to develop self-care skills and to evaluate and adjust drug therapy. However, it was also revealed that the service was not accessible for all of these patients, as some lacked the abilities and capabilities necessary, such as sufficient digital and cognitive knowledge to use the application or adequate Swedish language skills to take part in communication. This generated feelings of telemonitoring being less valuable.

When expectations and professional values of providing fair care are not in line with what an eHealth service offers, this may result in value destruction [20, 32]. The differences in abilities and capabilities described among patients may result in the emergence of health inequities [42], which would contradict the goal of Swedish care that care provision should be based on assessment of each patient’s individual needs and be made available to all patients on equal terms [50]. Therefore, it is necessary to address ethical and social dilemmas in order to promote health equity when using eHealth [41]. In line with the Swedish primary care reform “Good quality, local health care” [51], which states that the role of primary care should be strengthened, eHealth contributes to achieving the goal of reducing inpatient care and providing care in patient homes.

From a societal perspective, this raises the critical question of understanding which patients would gain the most value from using a telemonitoring service, which should be considered when deciding who should be entitled to take part. On the other hand, what is valuable for the individual patient may not always match what is valuable to society. It might be that the patients who are most chronically ill would have the greatest benefit. However, in order to use the telemonitoring application and devices, patients cannot be too ill, meaning that the technology may need to be simplified to better match their preconditions. It must be kept in mind that each patient is unique and has unique needs and preferences, which affects how an application should be designed, given that value is experienced uniquely and contextually [22, 24]. Having patients use the service solely for the value of trust and convenience, regardless of medical cost-effectiveness, may not be sustainable over time or even desirable. Furthermore, maximizing each patient’s subjective experience is not a purpose of publicly financed care. Private solutions provided outside the public health care system could potentially fulfil such needs, as digital services do not have geographical boundaries – but this would increase the risk of creating inequalities.

An important strength of this study was that it focused on the HCPs’ perspectives. Value research has previously been dominated by the patient perspective [15, 52–54] and it is considered of great relevance to investigate the experiences of HCPs, as value formation processes require interaction between many parties [19]. Although the sample size was small, the interview data were rich and the participants described their various experiences of how the eHealth application could function as a value-creating resource. Because this study took place during the COVID-19 pandemic, the interviews were performed at a distance, which might be a limitation. On the other hand, according to Archibald et al. [55], distance interviewing can be preferred over face-to-face interviews and highly satisfactory for the participants. We have described the sample, setting, data collection, and data analysis in detail to enhance transferability of the results to other groups or settings [56]. Finally, the findings may be of importance when planning to implement eHealth technology in similar contexts. However, to improve the understanding of value formation processes in eHealth, further research would benefit from exploring patients’ and HCPs’ experiences of their dyadic relationship.

## Conclusions

This study contributes by extending conceptualizations of value to the role of the health care professionals and by highlighting the dynamic role of technology in value formation processes. The findings indicate that the eHealth application was considered to be a value-creating resource through its facilitation of proactive communication as well as its support of patient engagement and control over self-care. The results describe the complexities and challenges in the value formation process when patients and HCPs used the telemonitoring application and devices. The findings indicate that it is important to have sufficient preconditions that enable interactive communication between parties, which may lead to creation of value. Absent these preconditions, value may be destroyed for at least one of the parties involved. Therefore, it is important that technology supports value creation. The nurses generally need better access to technical support and some need more time to be able to work successfully with the application. The proactive role of the HCPs and their over all value experience could be increased by having HCPs who work outside office hours with supporting patients through the application. Another important aspect is to develop the application to better meet patients’ individual needs and preconditions so that those in need will be able to use the application and gain better access to care.

## Data Availability

The interview data that were collected and analyzed in this manuscript are not publicly available due to participants not having consented to public availability. Aggregated data in Swedish are available from the corresponding author on reasonable request.

## Abbreviations

FLM: First-line manager
HCC: Health care center
HCP: Health care professional

## References

[1] Muka T, Imo D, Jaspers L, Colpani V, Chaker L, van der Lee SJ (2015). The global impact of non-communicable diseases on healthcare spending and national income: a systematic review. Eur J Epidemiol.

[2] Reynolds R, Dennis S, Hasan I, Slewa J, Chen W, Tian D (2018). A systematic review of chronic disease management interventions in primary care. BMC Fam Pract.

[3] Schiøtz ML, Høst D, Frølich A (2016). Involving patients with multimorbidity in service planning: perspectives on continuity and care coordination. J Comorbidity.

[4] Shrestha SS, Zhang P, Hora I, Geiss LS, Luman ET, Gregg EW (2019). Factors contributing to increases in diabetes-related preventable hospitalization costs among U.S. adults during 2001–2014. Diabetes Care.

[5] WHO. eHealth at WHO. https://www.who.int/ehealth/about/en. Accessed 1 Jun 2021.

[6] Elbert NJ, Van Os-Medendorp H, Van WilcoRenselaar, Ekeland AG, Hakkaart-Van Roijen L, Raat H (2014). Effectiveness and cost-effectiveness of ehealth interventions in somatic diseases: a systematic review of systematic reviews and meta-analyses. J Med Internet Res.

[7] Greenwood DA, Gee PM, Fatkin KJ, Peeples M (2017). A systematic review of reviews evaluating technology-enabled diabetes self-management education and support. J Diabetes Sci Technol.

[8] Koehler F, Koehler K, Deckwart O, Prescher S, Wegscheider K, Kirwan BA (2018). Efficacy of telemedical interventional management in patients with heart failure (TIM-HF2): a randomised, controlled, parallel-group, unmasked trial. Lancet.

[9] Kreps GL, Neuhauser L (2010). New directions in eHealth communication: opportunities and challenges. Patient Educ Couns.

[10] Lindberg B, Nilsson C, Zotterman D, Söderberg S, Skär L (2013). Using information and communication technology in home care for communication between patients, family members, and healthcare professionals: a systematic review. Int J Telemed Appl.

[11] Mann DM, Chen J, Chunara R, Testa PA, Nov O (2020). COVID-19 transforms health care through telemedicine: evidence from the field. J Am Med Informatics Assoc.

[12] Hägglund E, Strömberg A, Hagerman I, Lyngå P (2019). Theory testing of patient perspectives using a mobile health technology system in heart failure self-care. J Cardiovasc Nurs.

[13] McBain H, Shipley M, Newman S (2015). The impact of self-monitoring in chronic illness on healthcare utilisation: a systematic review of reviews. BMC Health Serv Res.

[14] McColl-Kennedy JR, Snyder H, Elg M, Witell L, Helkkula A, Hogan SJ (2017). The changing role of the health care customer: review, synthesis and research agenda. J Serv Manag.

[15] McColl-Kennedy JR, Vargo SL, Dagger TS, Sweeney JC, van Kasteren Y (2012). Health care customer value cocreation practice styles. J Serv Res..

[16] Batalden M, Batalden P, Margolis P, Seid M, Armstrong G, Opipari-Arrigan L (2016). Coproduction of healthcare service. BMJ Qual Saf..

[17] Gallan AS, Jarvis CB, Brown SW, Bitner MJ (2013). Customer positivity and participation in services: an empirical test in a health care context. J Acad Mark Sci.

[18] Palumbo R (2016). Contextualizing co-production of health care: a systematic literature review. Int J Public Sect Manag.

[19] Osei-Frimpong K, Owusu-Frimpong N (2017). Value co-creation in health care: a phenomenological examination of the doctor-patient encounter. J Nonprofit Public Sect Mark.

[20] Echeverri P, Skålén P (2021). Value co-destruction: review and conceptualization of interactive value formation. Mark Theory.

[21] Vargo SL, Lusch RF (2008). Service-dominant logic: continuing the evolution. J Acad Mark Sci.

[22] Chandler JD, Vargo SL (2011). Contextualization and value-in-context: how context frames exchange. Mark Theory.

[23] Spanjol J, Cui AS, Nakata C, Sharp LK, Crawford SY, Xiao Y (2015). Co-production of prolonged, complex, and negative services: an examination of medication adherence in chronically ill individuals. J Serv Res.

[24] Vargo SL, Maglio PP, Akaka MA (2008). On value and value co-creation: a service systems and service logic perspective. Eur Manag J..

[25] Spaling MA, Currie K, Strachan PH, Harkness K, Clark AM (2015). Improving support for heart failure patients: a systematic review to understand patients’ perspectives on self-care. J Adv Nurs.

[26] Lie SS, Karlsen B, Graue M, Oftedal B (2019). The influence of an eHealth intervention for adults with type 2 diabetes on the patient–nurse relationship: a qualitative study. Scand J Caring Sci.

[27] McCormack B, McCance TV (2006). Development of a framework for person-centred nursing. J Adv Nurs.

[28] Ekman I, Swedberg K, Taft C, Lindseth A, Norberg A, Brink E (2011). Person-centered care - ready for prime time. Eur J Cardiovasc Nurs.

[29] Vargo SL, Lusch RF (2016). Institutions and axioms: an extension and update of service-dominant logic. J Acad Mark Sci.

[30] Spanò R, Di Paola N, Bova M, Barbarino A (2018). Value co-creation in healthcare: evidence from innovative therapeutic alternatives for hereditary angioedema. BMC Health Serv Res.

[31] Payne AF, Storbacka K, Frow P (2008). Managing the co-creation of value. J Acad Mark Sci.

[32] Plé L, Chumpitaz Cáceres R (2010). Not always co-creation: introducing interactional co-destruction of value in service-dominant logic. J Serv Mark.

[33] Cajita MI, Gleason KT, Han HR (2016). A systematic review of mhealth-based heart failure interventions. J Cardiovasc Nurs.

[34] Robertson N, Polonsky M, McQuilken L (2014). Are my symptoms serious Dr Google? A resource-based typology of value co-destruction in online self-diagnosis. Australas Mark J.

[35] Engen M, Fransson M, Quist J, Skålén P (2021). Continuing the development of the public service logic: a study of value co-destruction in public services. Public Manag Rev.

[36] Huniche L, Dinesen B, Nielsen C, Grann O, Toft E (2013). Patients’ use of self-monitored readings for managing everyday life with COPD: a qualitative study. Telemed e-Health.

[37] Palumbo R, Manna R (2018). What if things go wrong in co-producing health services? Exploring the implementation problems of health care co-production. Policy Soc..

[38] Echeverri P, Skålén P (2011). Co-creation and co-destruction: a practice-theory based study of interactive value formation. Mark Theory.

[39] Öberg U, Orre CJ, Isaksson U, Schimmer R, Larsson H, Hörnsten Å (2018). Swedish primary healthcare nurses’ perceptions of using digital eHealth services in support of patient self-management. Scand J Caring Sci.

[40] Ministry of Health and Social Affairs and Swedish Association of Local Authorities. Vision for eHealth 2025-common starting points for digitisation of social services and health care. 2016. https://www.government.se/4a3e02/contentassets/b0fd09051c6c4af59c8e33a3e71fff24/vision-for-ehealth-2025.pdf. Accessed 3 Jun 2021.

[41] Hellberg S, Johansson P (2017). eHealth strategies and platforms – the issue of health equity in Sweden. Heal Policy Technol.

[42] Visser LM, Benschop YWM, Bleijenbergh IL, van Riel ACR (2019). Unequal consumers: consumerist healthcare technologies and their creation of new inequalities. Organ Stud.

[43] Varsi C, Ekstedt M, Gammon D, Børøsund E, Ruland CM (2015). Middle managers’ experiences and role in implementing an interactive tailored patient assessment eHealth intervention in clinical practice. CIN - Comput Informatics Nurs.

[44] Graneheim UH, Lundman B (2004). Qualitative content analysis in nursing research: concepts, procedures and measures to achieve trustworthiness. Nurse Educ Today.

[45] Krippendorff K. Content analysis: an introduction to its methodology. 3rd ed. Thousand Oaks: SAGE Publications; 2013.

[46] Zainuddin N, Tam L, McCosker A (2016). Serving yourself: value self-creation in health care service. J Serv Mark.

[47] Aiken LH, Sloane DM, Bruyneel L, Van den Heede K, Sermeus W (2013). Nurses’ reports of working conditions and hospital quality of care in 12 countries in Europe. Int J Nurs Stud.

[48] Hagerman H, Högberg H, Skytt B, Wadensten B, Engström M (2017). Empowerment and performance of managers and subordinates in elderly care: a longitudinal and multilevel study. J Nurs Manag.

[49] Dong B, Evans KR, Zou S (2008). The effects of customer participation in co-created service recovery. J Acad Mark Sci.

[50] SFS (2017:30) Hälso- och sjukvårdslag. (The health and medical act). https://www.riksdagen.se/sv/dokument-lagar/dokument/svensk-forfattningssamling/halso--och-sjukvardslag_sfs-2017-30. Accessed 3 Jun 2021.

[51] Ministry of Health and Social Affairs. Good quality, local health care – a primary care reform (SOU2018:39). http://www.sou.gov.se/wp-content/uploads/2018/06/Summary-SOU-2018.39_Final2.pdf. Accessed 3 Jun 2021.

[52] Sweeney JC, Danaher TS, McColl-Kennedy JR (2015). Customer effort in value cocreation activities: improving quality of life and behavioral intentions of health care customers. J Serv Res.

[53] Go Jefferies J, Bishop S, Hibbert S (2021). Service innovation through resource integration: an empirical examination of co-created value using telehealth services. Public Policy Adm.

[54] Hardyman W, Kitchener M, Daunt KL (2019). What matters to me! User conceptions of value in specialist cancer care. Public Manag Rev.

[55] Archibald MM, Ambagtsheer RC, Casey MG, Lawless M. Using zoom videoconferencing for qualitative data collection: perceptions and experiences of researchers and participants. Int J Qual Methods. 2019;18. 10.1177/1609406919874596.

[56] Graneheim UH, Lindgren BM, Lundman B. Methodological challenges in qualitative content analysis: a discussion paper. Nurse Educ Today. 2017;56:29–34. 10.1016/j.nedt.2017.06.002. 10.1016/j.nedt.2017.06.002 28651100

[57] World Medical Association (2013). WMA declaration of Helsinki: ethical principles for medical research involving human subjects. JAMA.

